# Severe distributive shock, neutrophilic dermatosis, and ST-elevation myocardial infarction in the setting of azathioprine hypersensitivity syndrome

**DOI:** 10.1186/s13223-024-00906-7

**Published:** 2024-07-20

**Authors:** Samuel Su, Yu Ming Wang, Karver Zaborniak, Sate Hamza, Davinder S. Jassal, Marcus Blouw

**Affiliations:** 1 Section of Cardiology, Department of Internal Medicine, Rady Faculty of Health Sciences, University of Manitoba, Winnipeg, MB Canada; 2 Department of Internal Medicine, Rady Faculty of Health Sciences, University of Manitoba, Winnipeg, MB Canada; 3 Section of Allergy and Clinical Immunology, Department of Internal Medicine, Rady Faculty of Health Sciences, University of Manitoba, Winnipeg, MB Canada; 4 Department of Pathology, Rady Faculty of Health Sciences, University of Manitoba, Winnipeg, MB Canada; 5 Department of Physiology and Pathophysiology, Rady Faculty of Health Sciences, University of Manitoba, Winnipeg, MB Canada; 6 Department of Radiology, St. Boniface Hospital, University of Manitoba, Winnipeg, MB Canada; 7 Section of Critical Care, Department of Internal Medicine, Rady Faculty of Health Sciences, University of Manitoba, Winnipeg, MB Canada

**Keywords:** Azathioprine, Hypersensitivity, ST-elevation myocardial infarction

## Abstract

**Background:**

Azathioprine is a purine synthesis inhibitor used as an immunosuppressive therapy for many immune-mediated diseases. Azathioprine hypersensitivity reaction is a rare, life-threatening adverse reaction characterized by a range of multisystem manifestations including fever, abdominal pain, arthralgias, erythematous cutaneous eruption, acute renal failure, neutrophilia, and more rarely, distributive shock. Although acute heart failure has been rarely described in association with azathioprine hypersensitivity syndrome, myocardial infarction has, to our knowledge, never been associated with this entity.

**Case Presentation:**

We describe a case of a 59-year-old male with Crohn’s disease who developed severe azathioprine hypersensitivity syndrome that included distributive shock, neutrophilic dermatosis, and acute coronary syndrome with ST-elevation. Clinical improvement was seen after cessation of azathioprine and administration of glucocorticoid therapy.

**Conclusion:**

Prompt recognition of azathioprine hypersensitivity syndrome, which can manifest as shock and neutrophilic dermatosis, is key to ensure rapid azathioprine cessation.

**Supplementary Information:**

The online version contains supplementary material available at 10.1186/s13223-024-00906-7.

## Background

Azathioprine hypersensitivity syndrome (AHS) is a rare, life-threatening adverse reaction characterized by a range of adverse effects including fever, hypotension, abdominal pain, arthralgias, erythematous cutaneous eruption, acute renal failure, and neutrophilia [1]. It is thought to be mediated by a type III or IV hypersensitivity reaction, and/or from increased production of inflammatory cytokines [2–4]. Of the relatively few cases of AHS reported in the literature, [1–6] none have described concurrent ST-elevation myocardial infarction (STEMI). We describe a case of a 59-year-old male with fibrostenotic Crohn’s disease who developed severe AHS with therapy initiation and subsequently an acute coronary syndrome (ACS) upon a re-challenge with azathioprine.

## Case presentation

A 59-year-old man with longstanding fibrostenotic Crohn’s disease presented with a one-day history of fever and abdominal pain. One week prior to his hospital admission, he had a surveillance colonoscopy demonstrating progression of his Crohn’s disease, and therefore started on azathioprine 125 mg daily after routine testing of thiopurine methyltransferase (TPMT) levels were found to be intermediate at 38 nmol/g Hb/h (Intermediate: 10–40, Normal > 40). The patient’s medical history consisted of Crohn’s disease, iron deficiency, and benign prostatic hyperplasia. The patient did not have any known skin disease or allergies. The patient was on the following medications: Azathioprine 50 mg PO daily, Budesonide 3 mg PO daily, Tamsulosin 0.4 mg PO daily, and Ferrous Gluconate 300 mg PO daily. The patient was admitted to hospital with a working diagnosis of sepsis and was started on empiric parenteral ceftriaxone. Investigations including chest radiograph, blood and urine cultures, and computed tomography of the abdomen and pelvis revealed no source of infection. Azathioprine was held on admission due to concern of immunosuppression exacerbating an underlying infectious process.

**Fig. 1:**
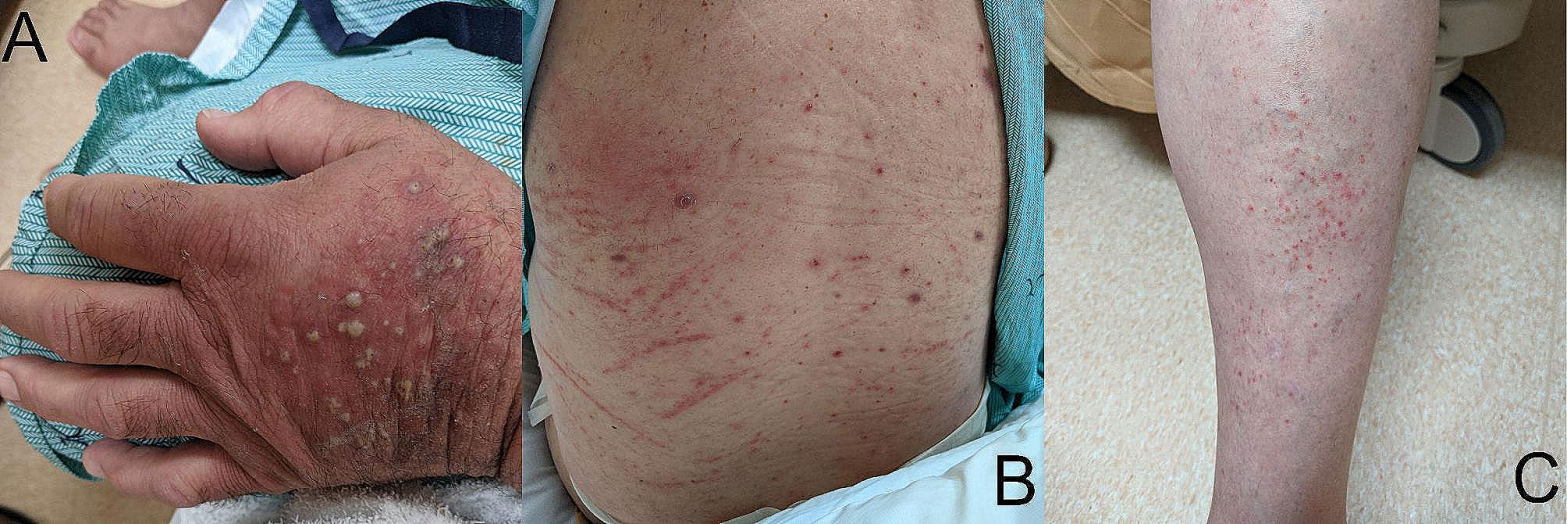
Cutaneous eruptions that developed in our reported case of AHS. (**A**) Dorsal aspect of left hand: Erythematous plaque with pustules, some papules, and patchy crusting. (**B**) Back: Patchy poorly demarcated erythema, irregularly distributed variably excoriated macules and papules, and linear scratch marks. (**C**) Left leg: multiple small erythematous non-blanching petechiae

Three days after admission, he continued to have persistent tachycardia and fever which progressed to distributive shock and acute renal failure. The patient received broad spectrum antimicrobial therapy with piperacillin-tazobactam and vancomycin, stress-dose methylprednisolone, and vasopressor support with norepinephrine for refractory shock. One day after his first day in the ICU and four days after admission to hospital, he developed an acute rash, predominantly on his extremities and torso (Fig. 1A-C). The rash was treated with topical steroid cream and a punch biopsy from the dorsal right hand was sent for pathological assessment.

With clinical improvement, and the decreasing concern for immunosuppression, the patient’s azathioprine was re-started on day seven of his ICU admission. Approximately twelve hours after receiving a single re-challenge dose of azathioprine, the patient developed recurrent vasodilatory shock and decline in level of consciousness, requiring endotracheal intubation, fluid resuscitation, and re-initiation of vasoactive medications. His electrocardiogram demonstrated transient ST elevation in the inferior and septal leads with an elevated serum high-sensitivity troponin T of 1290 ng/L (upper reference limit: 14 ng/L). Urgent cardiac catheterization revealed no evidence of obstructive coronary artery disease.

**Fig. 2:**
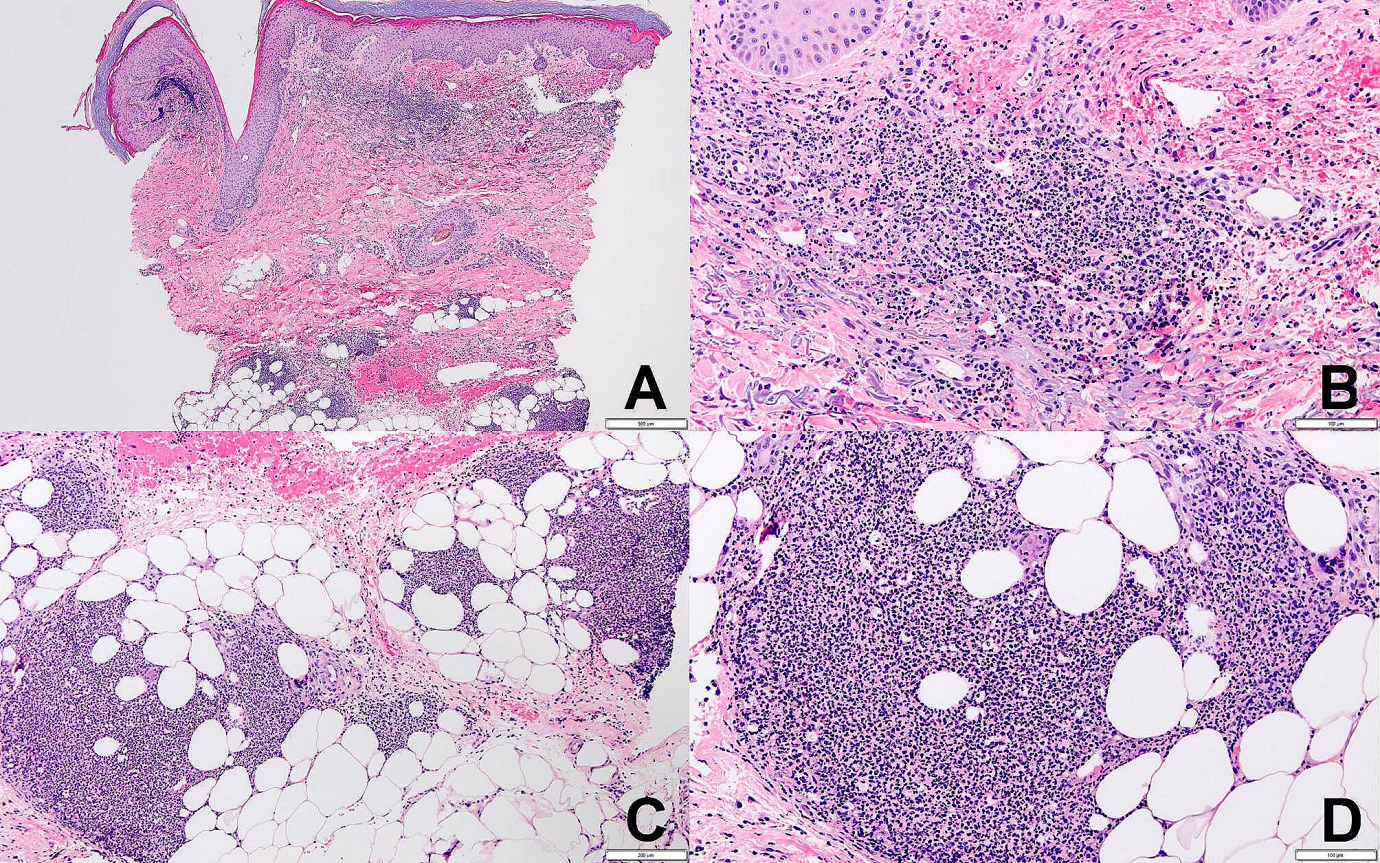
Skin punch biopsy from right hand dorsum demonstrating a patchy upper dermal and superficial subcutaneous inflammatory infiltrate composed of neutrophils. Extravasated red cells are also seen in the upper dermis (**A**, **B**). Note the karyorrhexis in the dermal infiltrate (**B**) and the microabscess formation in the subcutis (**C**, **D**) [Hematoxylin and Eosin. Original magnification X40 (**A**), X200 (**B**), X100 (**C**) and X200 (**D**)]

At this point, AHS was suspected to be the cause of the patient’s distributive shock given the timeline of recent initiation of azathioprine prior to presentation, improvement with cessation of the medication, and immediate clinical decline with a re-challenging dose. The results of the skin biopsy from the dorsum of the right hand (Fig. 2A-D) revealed a variable perivascular and diffuse mixed inflammatory infiltrate composed of neutrophils with mononuclear inflammatory cells and eosinophils present in the upper dermis, with upper dermal extravasation of red cells, and a neutrophilic infiltrate with microabscess formation present in the superficial subcutis. Based on clinicopathologic correlation, a diagnosis of neutrophilic dermatosis, consistent with AHS was rendered, alongside ACS with ST-elevation related to this drug reaction.

After identification of AHS as the most likely cause of the patient’s clinical syndrome, azathioprine was discontinued indefinitely, and immunosuppression was provided with systemic corticosteroids. The dermatologic abnormalities resolved completely over the following two weeks.

## Discussion

AHS is relatively rare, seen in only about 2% of patients initiating this immunosuppressive therapy [1, 2]. Adverse effects have been reported to occur between two and fourteen days after initiation of the medication [1–6]. Once a patient is sensitized, a re-challenge of the drug will typically have a rapid onset of the reaction, with previous case reports in the literature documenting onset within hours of re-exposure [1–6]. As supported by our case, the hypersensitivity reaction from re-exposure to azathioprine is often more severe, with hypotension and shock reported in up to one-third of cases [1–6]. The mainstay of treatment is supportive measures with fluid resuscitation and vasopressor/inotropic support if necessary. The hypersensitivity reaction typically resolves within days to weeks of cessation of the drug.

Azathioprine is metabolized to its active form 6-mercaptopurine (6-MP) and is subsequently inactivated by a methylation reaction catalyzed by thiopurine S-methyltransferase (TPMT). TMPT levels are commonly assessed prior to azathioprine initiation to prevent the side effects and toxicities that arise from azathioprine’s toxic metabolites (e.g. fevers, nausea/vomiting, granulocytopenia, and hepatocellular liver injury). Unlike the known mild to moderate toxicities of azathioprine that have been associated with low TPMT, azathioprine hypersensitivity has not been previously associated with TPMT levels.

Various rashes have been associated with AHS including erythema nodosum, small-vessel vasculitis, acute generalized exanthematous pustulosis (AGEP), nonspecific dermatitis, and Sweet syndrome. In the present case, the association of fever, neutrophilic dermatosis and of a severe systemic inflammatory response leading to shock and myocardial infarction (MI), could either be compatible with a diagnosis of drug-induced Sweet-like syndrome or Acute Generalised Exanthematous Pustulosis (AGEP), both of which having been previously described with azathioprine [1, 5]. Although systemic steroids are the mainstay of initial treatment in Sweet syndrome [7], they are not standard of care in AGEP [8]. Rather, in AGEP, resolution is typically noted within a short time frame after discontinuing the causative agent, and while topical steroids have been used to provide symptom relief, but the role of systemic steroids in AGEP is not well-established [8].

While the rash of neutrophilic dermatosis and distributive shock has been previously described in the setting of AHS, there have been no reports of azathioprine hypersensitivity and concurrent ACS. According to the 4th Universal Definition of Myocardial Infarction, the ACS in the case best fits the diagnosis of type II MI, which is defined as a “detection of a rise and/or fall of cardiac Troponin values with at least 1 value above the 99th percentile of the upper reference limit, and evidence of an imbalance between myocardial oxygen supply and demand unrelated to coronary atherothrombosis” with typical features of: symptoms of acute MI, new ischemic ECG changes, and/or imaging evidence of a new loss of viable myocardium or regional wall motion abnormalities in an ischemic territory. As such, in the absence of significant coronary atherosclerosis or coronary dissection, the ACS in the case was most likely a type II MI related to coronary vasospasm or oxygen supply-demand mismatch. Allergic acute coronary syndromes have previously been described in an entity known as Kounis syndrome, which is broadly defined as an ACS in the setting of a systemic allergic response, specifically related to vasospasm from inflammatory mediators in type I (IgE) hypersensitivity [9]. However, in the presented case, the mechanism of AHS is more related to massive systemic cytokine response relating to T cells and neutrophils, and therefore, does not quite meet previous definitions for Kounis syndrome. While inflammatory cytokines can play a role in atherothrombotic-related MI [10], this has not been well-established in non-atherothrombotic MI, as in the presented case. In the literature, little is known about ACS, including STEMI, in the context of cytokine release syndromes. While cytokine release syndromes have been recently postulated to be involved in COVID-19 infection-related acute coronary syndromes, the mechanisms are not well-established yet [11]. Further studies focused on the mechanisms of ACS in the setting of cytokine release syndromes, including azathioprine hypersensitivity, are required.

## Conclusions

Azathioprine hypersensitivity should be considered in the differential diagnosis of patients recently started on the medication presenting with a distributive shock and acute coronary syndrome. Cessation of azathioprine appears to be effective in resolving both reactions. Avoidance of azathioprine should be strongly recommended in patients who develop this severe reaction, especially given the availability of alternate immune-mediating therapies.

### Electronic supplementary material

Below is the link to the electronic supplementary material.

Supplementary Material 1

## Data Availability

No datasets were generated or analysed during the current study.
