# Supplementary material for: Severe distributive shock, neutrophilic dermatosis, and ST-elevation myocardial infarction in the setting of azathioprine hypersensitivity syndrome
Source: Allergy Asthma Clin Immunol. 2024 Jul 20;20:39. doi: 10.1186/s13223-024-00906-7 (PMC11264972; doi:10.1186/s13223-024-00906-7)
Supplement: Supplementary file 1 — Supplementary Material 1 [file 13223_2024_906_MOESM1_ESM.docx]

**Supplemental Table 1.** Detailed timeline of events described in the clinical case report.

| **Time** | **Event** |
| --- | --- |
| Day 0 | - Colonoscopy demonstrated progression of known Crohn’s disease and neoterminal ileitis. - Started on azathioprine (125mg daily). |
| Day 7 | - Presentation to the emergency room with fever and abdominal pain. - Admitted to medical ward and started on empiric ceftriaxone. - Azathioprine withheld on admission. - Cultures and abdominal CT did not reveal a source of infection. |
| Day 10 | - Worsening shock, acute renal failure. - Transferred to ICU. - Fluid resuscitation, vasopressor support. - Antimicrobials broadened to piperacillin-tazobactam and vancomycin. - Stress dose methylprednisolone. |
| Day 11 | - Assessment reveals multiple cutaneous eruptions (Figure 1). - Topical steroids applied with no benefit. |
| Days 12-16 | - Clinical improvement of shock, weaning of vasopressors. - Further microbiologic cultures negative. |
| Day 16 PM | - Azathioprine restarted. |
| Day 17 AM | - Recurrence of acute shock and decreased level of consciousness. - Electrocardiogram changes (Figure 2). - Re-initiation of vasopressor and inotropic medications. - No evidence of obstructive coronary disease on cardiac catheterization. |
| Day 17 PM | - Initiation of renal replacement therapy for renal failure. |
| Day 18 | - Suspected Azathioprine as cause of decompensation. - Stopped Azathioprine indefinitely. - Initiation of tapering corticosteroid course. |
| Day 19-22 | - Clinical improvement of shock, renal failure, weaning of vasopressors. - Further cultures continue to be negative. |
| Day 23 | - Transfer from ICU to medical ward. |
| Day 23-30. | - Gradual resolution of rash. - Weaning of renal replacement therapy. |
| Day 30-74 | - Prolonged rehabilitation course |
| Day 75 | - Discharge from hospital |

**Supplemental Table 2.** Laboratory values during the patient’s initial presentation compared to after the re-challenging dose of Azathioprine.

| **Lab Value** | **Initial Presentation**  (Day 7) | **After Re-Challenging Dose**  (Day 17) |
| --- | --- | --- |
| Hemoglobin (g/L) | 115 | 117 |
| White Blood Cells (x10^9^/L) | 16.6 | 45 |
| Platelets (x10^9^/L) | 176 | 450 |
| Creatinine (umol/L) | 120 | 720 |
| Troponin T (ng/L) | 70 | 1290 |
| AST (U/L) | 30 | 90 |
| ALT (U/L) | 45 | 32 |
| ALP (U/L) | 60 | 105 |
| GGT (U/L) | 90 | 150 |
| C-Reactive Protein (U/L) | - | 216 |
